# Implementation and acceptability of high efficiency particulate air filters to reduce respiratory infections in care homes: Process evaluation of the AFRI-c cluster randomised controlled trial

**DOI:** 10.1371/journal.pone.0347989

**Published:** 2026-07-27

**Authors:** Sophie Rees, Ruth Kipping, Rachel C. M. Brierley, Clare Clement, Nicholas Turner, Eleanor Gidman, Karen Sargent, Jane Sprackman, Alastair D. Hay

**Affiliations:** 1 Bristol Trials Centre, Bristol Medical School, University of Bristol, United Kingdom; 2 Centre for Public Health, Bristol Medical School: Population Health Sciences, University of Bristol, United Kingdom; 3 Centre for Appearance Research, University of West of England, United Kingdom; 4 Centre for Academic Primary Care, Bristol Medical School: Population Health Sciences, University of Bristol, United Kingdom; U.S. Food and Drug Administration, UNITED STATES OF AMERICA

## Abstract

Respiratory infections are easily transmitted within care homes. Within a clinical trial (called AFRI-c), which tested the effectiveness of high-efficiency-particulate-air (HEPA) filters to reduce respiratory infections in care home residents, we conducted a mixed-methods process evaluation. We aimed to understand their acceptability, fidelity, and implementation to aid interpretation of the effectiveness findings. We used qualitative remote and face-to-face interviews with staff (n = 25), residents (n = 20), and relatives (n = 12) from care homes (n = 22) in the AFRI-c trial. We purposively sampled homes for variation in size, nursing or residential provision, and deprivation. We used reflexive thematic analysis, drawing on normalisation process theory to understand implementation. We used staff questionnaires (n ranges from 191 to 351 depending on questionnaire) and resident (n = 1158) questionnaires with descriptive and regression analyses. We used the triangulation protocol to integrate qualitative and quantitative findings. The use of HEPA filters became normalised, although some residents disliked the draught. Self-reported intervention fidelity was high, which is important context for interpreting the trial’s null outcome for infection reduction. HEPA filters made no difference to resident or staff satisfaction with the care home environment. We found no evidence that using HEPA filters changed infection control and prevention practices. While staff felt it was a priority to prevent respiratory infections, residents were more concerned about quality of life and care. Our mixed methods process evaluation of the AFRI-c trial found the use of HEPA filters was acceptable, with high levels of adherence and low levels of contamination, suggesting that the null trial results were not due to poor adherence. Some effort was required to ensure they were kept on. Approaches to data collection may have caused under-reporting of mild infections. We should not assume that infection prevention is always a priority for residents.

## Introduction

Respiratory infections are a leading cause of morbidity and mortality for older people living in residential care settings [1,2]. Respiratory infections, such as influenza and SARS-Cov-2 spread through direct contact, droplets depositing directly onto another person, and airborne transmission [3]. The care home setting makes it challenging to prevent spread of infections due to high levels of interaction between care home staff, residents, and visitors, and the logistical difficulties of isolating residents [2]. One potential way to reduce airborne transmission is the use of high-efficiency-particulate-air (HEPA) filters, which are known to remove airborne microbes [4]. However, there is an absence of evidence regarding whether this results in fewer symptomatic infections [5,6]. The ‘Air Filtration to prevent symptomatic winter Respiratory Infections (including COVID-19) in care homes’ (AFRI-c) was a cluster randomised controlled trial (RCT). It investigated the clinical- and cost-effectiveness of portable HEPA filters in resident and communal rooms to reduce respiratory infection episodes in older care home residents [7].

Introducing a new intervention into the dynamic and complex social environment of a care home, which involves various stakeholders, including residents, requires careful consideration of the contextual factors that may influence the intervention [8]. These factors include the level of support from the care home management team, how well the research processes fit with existing workloads, and the level of engagement required to deliver the intervention [8]. Process evaluation methodology is used to assess fidelity, acceptability, and implementation of interventions within a trial [9,10]. In the AFRI-c trial, which found no evidence that HEPA filters reduced infections during winter months [11], this process evaluation aimed to interpret the trial findings. We explored the acceptability, fidelity, and implementation of the intervention and AFRI-c trial processes, including data collection at site level, and perceptions of the HEPA filters. This helped us to better understand how contextual factors, such as support from care home management, alignment with existing workloads, and engagement levels, may have influenced the trial’s outcome.

## Materials and methods

### Design and aims

This study was a mixed-methods process evaluation of the AFRI-C trial and intervention. AFRI-c was a cluster RCT (trial registration ISRCTN63437172), where 91 care homes in England were recruited and randomised to either usual care (n = 44) or use of HEPA filters (n = 47). The intervention care homes received portable HEPA filters for use in up to five communal areas and between 10–16 bedrooms for September to May. The trial took place over three winters between September 2021 to May 2024. Full details of the trial are available in the published protocol [7].

The aims of the AFRI-c mixed methods process evaluation were:

- to understand acceptability, fidelity, and implementation of the AFRI-c intervention
- to contribute to the interpretation of the clinical effectiveness findings.

**Fig 1:**
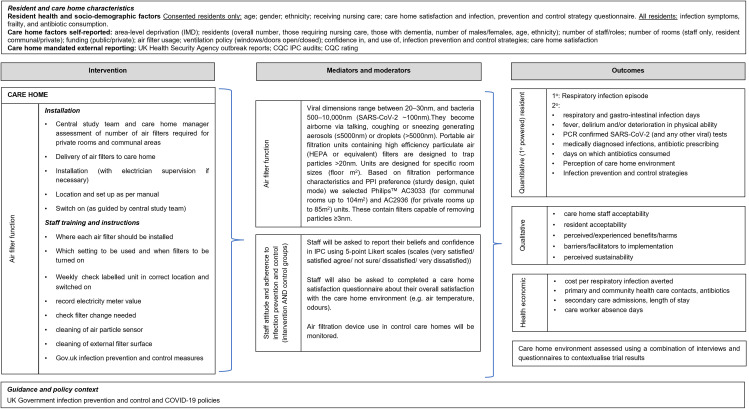
AFRI-c process evaluation logic model.

**Fig 2:**
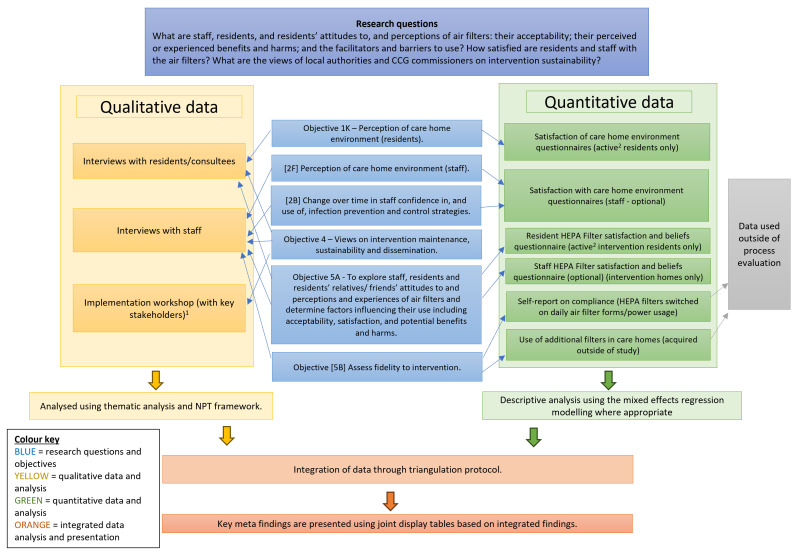
AFRI-c process evaluation objectives and data. ^1^Not carried out due to lack of clinical effectiveness findings, ^2^Active residents are defined as the 10-16 per home selected to have symptoms monitored daily and/or a bedroom HEPA filter (intervention only).

We developed a logic model (Fig 1) to define the components of the intervention, the mediators and moderators, and the outcomes by which they would be determined, as well as the relevant overall policy context. We also plotted these outcomes against our objectives to identify the data that would contribute to each objective (Fig 2). We used Normalisation Process Theory (NPT) [12] to aid understanding of intervention implementation. NPT is a social theory used in implementation research to help understand the work required from actors in a social context to implement and deliver an intervention or practice. NPT has four constructs: coherence (understanding the purpose of an intervention), cognitive participation (engagement with the intervention), collaborative action (steps involved to use an intervention), and reflexive monitoring (appraisal of an intervention).

### Qualitative component

#### Participants.

Participants in the qualitative study were: care home staff involved in delivering AFRI-c (intervention and control care homes); residents with capacity who had a HEPA filter in their bedrooms (intervention care homes); relatives who acted as personal consultees for residents without capacity who had a HEPA filter in their bedrooms (intervention care homes).

#### Interviewee recruitment and sampling.

The recruitment process involved a systematic approach to identify potential participants across the three groups: care home staff, consultees and residents. The AFRI-c trial recruitment commenced on 01/02/2022 and completed on 31/05/2024. Qualitative data were collected during: 23/03/2022 to 30/5/2022 (Winter 1), 23/11/2022 to 06/04/2023 (Winter 2), and 09/02/2024 to 04/04/2024 (Winter 3).

Staff participants: In Winter 1, we used convenience sampling and approached staff in all six care homes in the intervention arm of the study. In Winters 2 and 3 we used maximum variation purposive sampling of sites, to gain a sample of care homes diverse in terms of: care home size (number of residents); regional location; Index of Multiple Deprivation (IMD) and trial arm (intervention or control). The researcher (SR) directly approached potential participants. Using trial records, study champions (the main study contact within the home) were contacted and asked to identify the most appropriate staff member(s) for interview.

Resident participants: Residents and consultees who consented to the trial had indicated their willingness to be contacted about an interview in a written consent form at trial baseline and were identified using the trial consent database. To sample residents, we identified homes with high numbers of residents with capacity who had agreed to be approached about the interview, sampling for variation in the IMD of the care home. The researcher (SR) arranged the visit in advance, and upon arrival confirmed with the study champion whether it was appropriate to approach each named resident on that day.

Consultee participants: We purposively sampled to achieve diversity in age, gender, and ethnicity of the residents they represented. Before making contact, the researcher checked with study champions to ensure it was still appropriate to contact each consultee (e.g., residents not recently transferred to hospital, deteriorated, or passed away).

We emailed the relevant Participant Information Leaflet (PIL) to staff and consultees at the point of initial contact about the study. For residents, the PIL was provided by study champions at the care home prior to the scheduled visit. Study champions ensured residents understood we were visiting and that someone had explained the information to them before the visit.

#### Qualitative data collection.

Interviews took place at least four weeks following the commencement of trial data collection at each home, and typically between February and May. This allowed participants to reflect over many weeks of the trial and/or intervention at their care home. Care home staff and consultees participated in either telephone or video call interviews, and verbal consent was recorded at the start of each interview. Verbal consent was also audio-recorded for resident interviews, which all took place face-to-face in the care homes, usually in the resident’s bedroom. All interviews were conducted by SR, except for one staff interview which was carried out by CC. All interviews were audio-recorded and professionally transcribed verbatim.

#### Qualitative data analysis.

Qualitative analysis techniques were applied at different stages of the trial to maximise data utility and inform and improve trial implementation as well informing more in-depth understanding of intervention implementation and interpretation of final trial findings. Analysis was supported by NVivo [13]. During Winter 1, SR and CC conducted initial rapid framework analysis [14] to share findings in a timely manner with the Trial Management Group and recommend changes to the trial protocol or communication with care homes for Winters 2 and 3. For example, we provided a ‘Top tips for preparing to be part of AFRI-c’ document at the start of Winter 2, which included lessons learned from interviewing staff in Winter 1. Following the initial rapid analysis, we analysed all qualitative data (from winters 1–3) using reflexive thematic analysis [15,16], informed by the normalisation process theory constructs, to aid interpretation of implementation themes. We conducted analysis alongside data collection and used preliminary findings to inform subsequent winter sampling and data collection (e.g., findings from Winter 2 were used to inform data collection during Winter 3). We coded interviews from Winters 1 and 2 initially using an inductive approach. CC and SR coded a sample of transcripts, and resultant codes and themes were discussed. SR and RK flexibly applied evolving codes and themes to Winter 3 transcripts and further developed them using Winter 3 data.

We edited the topic guide before Winter 3 interviews began, based on our initial coding from Winters 1 and 2. For example, we had enough data about study setup challenges and focused more on the intervention delivery itself. We added in further prompts around staff approach to data collection, and future intervention implementation. For consultees we added prompts about their worries regarding infections and how this related to the COVID-19 pandemic. The resident topic guides were unchanged between Winters 2 and 3, although our experience of interviewing residents led us to take a very flexible approach to the topic guide due to high variability in how easily residents were able to engage with the conversation.

### Quantitative component

#### Participants and data collection.

Table 1 shows the quantitative data collected for the process evaluation. We used resident and staff questionnaires to collect quantitative data on infection prevention and control strategies, satisfaction with the care home environment and HEPA filters, and compliance with intervention (staff-reported data collected daily on whether each HEPA filter was in position and switched on). Respondents to the questionnaires and case report forms were residents/consultees and/or staff at intervention or control sites. Staff questionnaires were all optional, given that staff are pressed for time, and there is often high turnover. Where a resident in the study lacked capacity, their personal consultee was invited to complete their questionnaire instead.

**Table 1:** Quantitative data collection.

Questionnaire/Case report form	Respondent type	Frequency
Confidence with infection prevention control strategies	Staff^1^	Baseline and end of Winter period
Beliefs about infection	Residents/consultees^2^ and staff	Baseline and end of Winter period
Care home environment satisfaction	Residents/consultees and staff	Baseline and end of Winter period
HEPA filter satisfaction and beliefs	Residents/consultees and staff (intervention only)	Baseline and end of Winter period
Self-report on HEPA filters (switched on and in correct location)	Staff	Daily
Use of additional HEPA filters in care home bedroom	Staff	Daily

^1^Staff questionnaires were optional; ^2^Where a resident lacked capacity, their personal consultee were invited to complete the questionnaire

#### Quantitative data analysis.

Staff questionnaires, resident/consultee belief in infection transmission, resident/consultee belief in effectiveness of air filters, resident/consultee sleep quality satisfaction, HEPA filter satisfaction and use of additional HEPA filters in resident bedrooms were analysed descriptively.

The 5-point Likert scale resident survey questions on perception of care home environment were formally compared between groups using mixed effects ordinal logistic regression methodology. The 5-point Likert scale for resident satisfaction questions had the response options of: very satisfied, satisfied, not sure, dissatisfied and very dissatisfied. All models were adjusted for care home nursing provision, socio-economic status (care home IMD), winter, and baseline survey responses as fixed effects and included a random effect to account for clustering by care home.

Data about the HEPA filter use (switched on and in the correct location) was reported descriptively. For participants with HEPA filters in their bedrooms, compliance with the trial intervention was defined per day as the bedroom air filter and at least three communal air filters being on. For participants without HEPA filters in their bedrooms compliance per day was defined as at least 3 communal air filters being on. Both continuous (percentage of days HEPA filter in use) and binary (HEPA filter in use at > 20% of checks) compliance was calculated.

### Mixed methods data integration

We used the triangulation protocol [17,18] to integrate the qualitative and quantitative data and findings, and to develop an overall interpretation to enhance the validity and depth of findings. This involved analysing the qualitative and quantitative datasets separately and then plotting key findings from each dataset in a joint-display matrix (Table 5) and identifying meta-themes that cut across the findings. Then each meta-theme was assessed against four categories: agreement, partial agreement, dissonance, and silence. Agreement represents convergence in the data, partial agreement reflects complementarity across data sets, dissonance represents conflicting findings, and silence reflects instances where findings were present in some data sets but absent from others.

It should be noted that staff were interviewed from both intervention and control homes because we wished to explore practices of data collection, whereas consultees and resident were only sampled from intervention care homes as interviews focused on experience of the intervention. Quantitative cross-sectional questionnaire data were collected from both intervention and control homes to enable comparisons to be made.

### Ethical approval

Ethical approval was given by London Harrow National Health Service (NHS) Research Ethics Committee (Ref 21/HRA/4318).

### Patient and public involvement (PPI)

Our PPI co-investigators (JS and KS) were instrumental in the development and refinement of the resident and consultee topic guides. For example, they recommended we break down the residents’ topic guide into higher and lower priority questions to accommodate any residents who may become tired during the interview. They also helped us to craft the questions to focus more on the present, so that they would be less likely to cause residents to become frustrated or feel unable to answer (for example if they could not remember something clearly).

## Results

### Participants

S1 Table (Supplementary data) shows *care home* characteristics (n = 22) and S2 Table shows *participant* (n = 57) characteristics. With COVID-19 pandemic restrictions in place during Winter 1, remote interviews (telephone/video call) with residents were attempted but were unsuccessful as residents either had poor hearing or found it difficult to interact over video call. A planned visit to conduct resident interviews was cancelled at short notice due to a COVID-19 outbreak in the home, and as a result we were unable to collect any resident data in Winter 1. Optional anonymised staff questionnaires which could be completed at baseline and end of Winter period were completed by staff members, with respondent numbers varying by questionnaire (all results shown in Tables 2–4 and in S1-S12 Tables). S3 Table shows the characteristics of all active residents who gave consent (or were included on advice of a consultee) for AFRI-c. Active residents were those who had valid daily data collection, where valid data was defined as collected daily data excluding days where a resident’s data was not required or a resident was not in the care home.

**Table 2:** Resident/consultee^1^ and staff satisfaction with HEPA filters (Intervention arm only).

	Residents	Staff
Very satisfied	**155 (42.6%)**	**66 (36.3%)**
Satisfied	**137 (37.6%)**	**71 (39.0%)**
Not sure	**65 (17.9%)**	**37 (20.3%)**
Dissatisfied	**6 (1.6%)**	**8 (4.4%)**
Very dissatisfied	**1 (0.3%)**	**0**
**Overall**	**364 (100%)**	**182 (100%)**

^1^Where a resident lacked capacity, a consultee was invited to complete the questionnaire on their behalf

**Table 3:** Resident/consultee^1^ satisfaction with care home temperature.

	Intervention	Control		
	n	n	Estimated OR^2^	95% CI
**Satisfaction with care home temperature**	356	393	1.00	0.63, 1.61

^1^Where a resident lacked capacity, their consultee was invited to complete the questionnaire; ^2^Estimated via mixed ordinal logistic regression

**Table 4:** Resident/consultee satisfaction with care home odour and air quality.

	Intervention	Control		
	n	n	Estimated OR^*^	95% CI
**Satisfaction with care home odour**	354	393	0.77	0.47, 1.24
**Satisfaction with care home air quality**	356	393	0.71	0.47, 1.08

* Lower odds ratios favour intervention homes; estimated via mixed ordinal logistic regression

### Triangulation of findings

A total of four meta-themes were developed through the triangulation protocol (Table 5) with insights from both qualitative and quantitative datasets. The results are structured around these meta-themes, illustrating key aspects from each dataset. These are: Intervention acceptability; Perceived intervention effectiveness; Intervention fidelity; and Trial data collection practices.

**Table 5:** Joint-display matrix for data integration.

Meta theme	Quantitative category/Finding	Qualitative code/Finding	Convergence category^1^	Conclusion
**Intervention acceptability**	Staff and resident satisfaction with HEPA filters was very high. Residents were generally satisfied with care home environment and sleep quality and there wasn’t much change or difference between the control and intervention groups. There was a trend that residents at intervention sites were more satisfied with odour and air quality in the care home environment but not statistically significant.	Staff, residents, and consultees generally found the air filters to be acceptable in the setting, although there were concerns about cold air when positioned too close to beds/chairs, especially the larger units. However there was also a sense that they ‘forgot’ they were there, indicating high acceptance. Staff and residents/consultees had different priorities, with residents prioritising quality of life and care over technology to prevent infections.	Partial agreement	There was high acceptance across both datasets, but we might have expected temperature to be a bigger issue in the quantitative data based on the qualitative data. Additionally, the findings around different stakeholder priorities were not explored in the quantitative data.
**Perceived intervention effectiveness**	At baseline, generally respondents felt HEPA filters would reduce infections, although residents were less sure than staff. Staff in the intervention arm appeared to become more polarised, with fewer ‘Not sure’ responses at follow-up. There was also a trend that residents in the intervention arm felt less strongly that HEPA filters would reduce infections at follow-up.	Staff appraised the effectiveness intervention based on their perceptions of infection rates, odour, and air quality. Additionally activity of the HEPA filters (e.g., debris in filter, noise) were seen as indicators of their effectiveness. Opinion was divided. Some staff felt ‘safer’ in areas with filters due to these perceptions.	Partial agreement	In both datasets, opinion was divided over perceived effectiveness. The findings about a sense of feeling safer in rooms with HEPA filters was not explored in the quantitative data.
**Intervention implementation: fidelity**	Self-reported compliance was very high. Data indicates some use of ‘non-trial’ HEPA filters in bedrooms of residents in intervention care homes, but only a small percentage of days were reported. There was no difference in confidence in infection prevention and control strategies between baseline-follow up or control-intervention groups.	Sometimes residents or staff switched HEPA filters off. Signs and reminders helped this to stop happening. Staff described continuing with usual infection prevention and control strategies.	Partial agreement	Self-reported compliance was very high, but in qualitative data participants reported incidents of HEPA filters being switched off, and the need for reminders. There did not seem to be any evidence of changes in infection prevention and control strategies in either sources of data.
**Trial implementation: data collection practices**	N/A	Staff described getting info from online care systems, as well as handovers and individually checking with each resident.	Silence	Silence because we did not explore this in the quantitative data.

^1^Agreement represents convergence in the data, partial agreement reflects complementarity across data sets, dissonance represents conflicting findings, and silence reflects instances where findings were present in some data sets but absent from others.

### Intervention acceptability

#### Impact on the care home environment.

Staff and consultees reflected that before the HEPA filters were installed, they had concerns about how ‘intrusive’ they would be (e.g., noisy, flashing lights, and space taken up in the room). However, once installed, participants mostly felt that the units were quiet and unobtrusive, and they ‘forgot’ they were there.

*I think most of the staff forget they’re there now…Yeah, they all seem to be quite happy with it. They’re not really in the way, so it’s not really something that they notice that often.* – 035, Winter 2 (W2) Staff, Intervention, Site L

*They’re part of our lives now!...We haven’t had a complaint about it from any resident.* – 045, W3 Staff, Intervention, Site R

*It doesn’t really affect me at all, I don’t think about it.* – 028, W2 Resident, Site K

This suggests a high level of acceptability, and a low level of cognitive engagement with the intervention required once installed.

However, participants did sometimes describe that the machines gave off a cold air draught, and this was especially a problem for residents and consultees.

*It’s blimmin’ cold*…*I don’t like that* – 026, W2 Resident, Site J

*In the communal areas a lot of them don’t like to sit right by it because either the lights are on or they can feel a bit of a breeze…we’ve tried to put it away from seating areas.* – 039, W2 Staff, Intervention, Site N

Questionnaire data showed that staff satisfaction with the HEPA filters was high (75% (n = 137/182) Very Satisfied or Satisfied (Table 2). Most residents/consultees with HEPA filters reported being very satisfied or satisfied (80%, n = 292/364). Satisfaction with sleep quality was similar in both the control (75% Very/Satisfied, n = 295/393) and intervention (80% Very Satisfied or Satisfied, n = 254/351) resident groups at follow-up, suggesting the HEPA filters did not tend to disturb their sleep (S4-S5 Table).

We performed a regression analysis of resident/consultee satisfaction with care home temperature, finding no evidence of a difference between the control and intervention (Table 3).

#### Differing stakeholder priorities.

Staff wanted to protect the health of residents, and they also perceived potential wider benefits such as reducing staff sickness and absenteeism, and preventing transfer of residents to hospital. As a result, they viewed infection prevention in the care home to be a high priority, and therefore welcomed any intervention that might support this, making the HEPA filters very acceptable to them in principle.

*In a care home, any kind of infections, especially respiratory tract infections can be deadly basically so it’s very important for us to keep those away from us as much as possible. –* 008, W1 Staff, Intervention, Site D

*It’s a better quality of life for residents, it can save on hospital admissions, save on antibiotics, doctors’ visits, even for the staff at work it could potentially decrease sickness within the staff team as well, so for those reasons it’s a great thing.* – 040, W3 Staff, Intervention, Site O

However, some consultees had a more nuanced or ambivalent position, recognising that preventing infection might not always be top priority for individual residents, but also acknowledging potential benefits to the care homes.

*If they’ve got a quality of life and they’re happy and enjoying themselves then preserving their life seems the right thing to do. But of course, the irony is sometimes an infection in a nursing home can be a kindness.* – 009, W1 Consultee, Site C

*If you were interviewing my stepmother, she’d be saying that. ‘Well, whatever’, you know, but I do think we have to look at the bigger picture and have them to protect everybody in that working environment.* - 044 W3 Consultee, Site P

Indeed, most of the residents we interviewed were unconcerned about infections, and sometimes expressed a sense of fatalism.

*My wife’s dead so I’m not worried about anything. The only thing that can happen to me is I can rest in peace with my wife, and I can’t wait for that day to come.* – 032*,* W2 Resident, Site K

*I’m 98 and I’m not immortal. I might catch something and depart, so what? I’ve got to go some day!* – 023, W2 Resident, Site J

*If I get a cold I get a cold. Hopefully I get over it. When you’re my age you stop worrying about things like that.* – 053, W3 Resident, Site U

One resident explicitly stated she *wanted* a chest infection as she was ready to die.

*I’d love a chest infection, I’m 96 I want to die. –* 059, W3 Resident, Site O

As a result, residents were largely indifferent to the idea of having air filters, and some felt that they were unnecessary in care homes, even if they were effective. Although this did not make the HEPA filters ‘unacceptable’, it did mean that residents were more concerned with, and would prioritise, their overall quality of life and care received from staff over technology to prevent infections.

*I’m happy here in this place to tell you the truth. People say a lot about care homes and that they’re horrible but good gracious me they can’t do enough for you. They help me…I wouldn’t fault anything…I’m quite happy here, with my lot*. – 024, W2 Resident, Site J

Similarly, when we explored further with consultees they told us that other aspects of the home, such as the caring nature of the staff, were more important than facilities provided by the home.

*I think depending on the cost I think if it wasn’t that much different, then if I thought they were working and that had been proved in the study, yeah, I would probably go for [home with an air filter] but I think overall from that is the quality of care that the home is offering.* – 043, W3 Consultee, Site Q

*Sometimes they can have nicer things, but it has to be what feels right…you can have all of that, but if the core foundation of it isn’t there then, it’s not worth it.* – 046, W3 Consultee, Site S

Our triangulation found *Partial agreement* for the ‘Intervention acceptability’ meta-theme, since the HEPA filters were considered broadly acceptable in both datasets (with some concerns around cold air), but in the qualitative data residents and consultees were more concerned about quality of life and care over infection prevention.

### Perceived intervention effectiveness

When appraising whether the HEPA filters were ‘working’, interviewees sometimes drew on their personal assessments of how many infection outbreaks they had experienced that winter. Their views on this were divided, with some feeling that there had been an improvement, and others feeling that the intervention had not made a difference.

*There’s been less incidents with residents of respiratory stuff, but there’s also been less ill health from staff.* – 037 W2 Staff, Intervention, Site M

*She’s probably only had…one cold. She’s also not had Covid, which has been brilliant. So, I think from that point of view, from stopping sort of airborne colds and anything that’s airborne, it must help because her room is not big.* – 006, W1 Consultee, Site B

*I would say over the last three weeks, I have probably thought ‘no [they are not working]’, because we have had quite a few residents with chest infections and viral infections and things like that. – 040, W3 Staff, Intervention*, Site O

*It hasn’t made any difference to me, I’m not aware of any difference*…*I think I’ve had a cold since it was here. –* 053, W3 Resident, Site U

However, people also drew on other experiences, such as a sense that there was a noticeable improvement of odour and general air quality within the home (‘freshness’).

*We’ve got one air purifier in the lounge and it’s very nice…When we are spending our time with [the residents], we can stay there and we can feel the difference you know…You can see it – the air’s very good and it doesn’t have the, you know – it’s fresh.* – 010, W1 Staff, Intervention, Site D

*Two of the consultees said that, whenever they come and visit their relative, they felt that the air is better…that’s what they said – ‘It feels different here, it’s nice.’* – 004, W1 Staff, Intervention, Site C

Other factors influenced their assessment of the effectiveness of the HEPA filters, such as seeing the dirt caught inside the filters, or witnessing its increased activity when there were more people present in a room.

*Having had to change filters on them, I realise how much debris is in the air that people are breathing in. So, until you actually see a filter that needs changing in front of you, you don’t realise how much general debris there is in the air that is being breathed in by people.* – 037, W2 Staff, Intervention, Site M

*[When] a lot of staff are there and we are talking, it increases the pressure, and then – amazingly – the colour changes…and then you can really hear that the pressure is more faster, and the number’s increasing and then, slowly and slowly, it will then settle down, and then, that’s the sign that it’s working*…*So, you can really tell that they’re purifying the air.* – 004, W1 Staff, Intervention, Site C

These visible/audible indicators of the HEPA filters’ activity reinforced staff’s confidents in the filters’ ability to purify the air and lower infection rates. This led some staff to describe feeling ‘safer’ with the air filters.

*The air feels cleaner. I don’t know whether it’s psychological, because you can see the filter doing its thing. –* 060, W3 Staff, Intervention, Site V

*There could be anything floating round in that room – someone could have coughed, someone could have sneezed – whereas you feel a lot cleaner going in a room with an air filter.* – 001, W1 Staff, Intervention, Site A

This was also supported by the cross-sectional quantitative data, which suggested that residents/consultees at the intervention sites may have been more satisfied with odour and air quality than those at control sites, however the confidence interval was wide and included the null (see Table 4).

At baseline, generally respondents felt HEPA filters would reduce infections, although residents/consultees were less sure than staff (S6 and S7 Tables). Little changed in the control arm between baseline and follow-up in the resident/consultee views. Staff in the intervention arm appeared to become more polarised over the course of the trial, with fewer ‘Not sure’ responses about whether the filters would reduce infection than at baseline, and an increase in the proportion of staff disagreeing that they would reduce infections at follow-up.

In both staff and resident/consultee responses, the vast majority agreed with the idea that infections could be transmitted through the air at baseline (in both intervention and control arm). There may have been a slight increase in people disagreeing with this between baseline and follow-up in the intervention group, but these were small numbers (S6 and S7 Tables).

The triangulation indicated *Partial agreement,* since both the qualitative and quantitative datasets showed a divided opinion on the effectiveness of the HEPA filters in terms of reducing infections and odour/air quality. The qualitative findings around feeling safer in areas with air filters were unexpected and were not explored in the quantitative data.

### Intervention fidelity

#### Keeping HEPA filters switched on.

Many care home residents have cognitive problems including dementia or confusion. Staff and consultees expressed concerns about, or reported incidents of, confused residents moving, or otherwise tampering with the HEPA filter settings.

*We only had two incidents where, unfortunately, a resident urinated in the air filter [and]…We had one that put it on his windowsill.* – 034, W2 Staff, Intervention, Site K

*We have got one client which goes around and turns the one off that he sits nearest to, but that’s only because he doesn’t like the electricity being spent. We try and explain to him – he has dementia, so we have to keep reminding him to leave it on!* – 035, W2 Staff, Intervention, Site L

Interviewees reported that sometimes staff switched off or moved the HEPA filters, either accidentally while cleaning the touchscreen surface, or to use its plug socket for another purpose. Stickers were provided by the study team for the plugs, but some sites created additional signs to place nearby.

*We’ve put massive signs above them on the wall saying, ‘Do not turn off, do not move them against the wall. Make sure they’re still 20cm apart.*’ – 003, W1 Staff, Intervention, Site A

*I think, sometimes, when the cleaners clean – dust over the top of it – I think they hit a button by mistake.* – 034, W2 Staff, Intervention, Site K

The quantitative data indicated very high self-reported compliance. Each day staff reported on whether the HEPA filters were switched on and in the right position. We defined ‘non-compliance’ as air filter being in use at <20% of expected checks. According to the data, both binary and continuous measures of compliance were very high (>96%) in both the bedrooms and communal areas over the three winters for the residents in intervention care homes (S9-S12 Tables).

Our triangulation of this data indicated *Partial agreement*, since the qualitative dataset highlighted challenges with compliance, and the effort needed to maintain it.

#### Use of other HEPA filters.

We asked staff to collect data on whether residents had a non-trial HEPA filter in their bedroom, to assess whether there was any contamination of the intervention. We considered that, if residents and/or their relatives were interested in the idea of HEPA filters to prevent infections, they might decide to purchase one for their/their relative’s bedroom even if they were in a control care home or were not selected to have one as part of the AFRI-c study.

We found some evidence of low-level contamination with ‘non-study’ HEPA filter use (S8 Table). Of residents who gave consent for the study (i.e., they consented to questionnaire completion, data collection from their medical records and (if in intervention care home) a bedroom HEPA filter), 3% (n = 36/1158) were recorded at some point as having a non-study HEPA filter in their bedroom. Of residents who were only included in the anonymous daily symptom data collection (and were not selected to have an air filter in their bedroom in intervention care homes), 11% (n = 213/1936) were recorded at some point as having a non-study HEPA filter in their bedroom. The proportion with non-trial HEPA filters was higher in the intervention arm (17.5%, n = 176/1004) than the control (4%, n = 37/932). However, the number of days these non-study HEPA filters were reported as ‘in use’ was very low (1.2%, n = 1466/127785 in the intervention arm of residents who did not have a trial HEPA filter) (S8 Table).

In qualitative interviews, only two staff members reported that a HEPA filter had been purchased for or by a resident at their home, and these were both from the same site, so may have been speaking about the same resident.

*I know a lady up on my floor has – that got put in last week but other than that, no, I don’t know anybody else.* – 011, W1 Staff, Intervention, Site D

*I had one family member who purchased one of these filters a while ago, just before we started the study actually. She’d seen the posters on the walls.* – 008 W1 Staff interview, intervention, Site D

In summary, we did not see large numbers of days where non-study HEPA filters were used in the intervention or control residents’ bedrooms, but we do not have data on whether these filters were actually study filters being used with other residents, inaccurate data recording, or real installation of alternative HEPA filters.

#### Impact of the intervention on infection control practices.

Intervention and control homes were asked to continue with their routine infection prevention and control practice. To assess fidelity to this, we explored if and how the intervention or study had impacted on these.

001 (W1 Staff, Intervention, Site A): *We probably talk about it [infection control] a lot more now… obviously, we’ll wash our hands and stuff ‘cause that’s routine every single day, working in a care home, but we never talked much about it before we got the air filters.*

Interviewer: What kind of things d’you think are being talked about that were just taken for granted before?

*001: All of the dirty air!*

Other than perhaps higher awareness of airborne infection, participants felt that the study had not changed their infection control practices at all.

*We haven’t like been like ‘oh the air filters are here, yes, it’ll all be fine’. It’s just an extra protection really, that’s what it’s feeling like. We’re just feeling like it’s an extra help…I’m not going to take my mask off because there’s an air filter on.* – 003, W1 Staff, Intervention, Site A

*I don’t say it’s affected our infection prevention and control ‘cause – apart from the fact, obviously, of cleaning the filter, then – it hasn’t really affected the way we normally worked anyway*. – 034, W2 Staff, Intervention, Site K

*No, no [nothing has changed], we’re pretty hot on infection control. We do a lot of cleaning anyway.* – 045, W3 Staff, Intervention, Site R

Infection control protocols changed during the study because of the easing of COVID restrictions. In Winter 1 and for some of Winter 2, care home staff were still using masks as part of their everyday routine. Staff participants reported that since COVID, staff were more likely to *choose* to wear a mask if they had a cold.

*If some staff they do have coughs and colds in the building they may choose to now wear a mask which is their own choice, rather than us telling them they have to. -* 040, W3 Staff, Intervention, Site O

*If staff have symptoms of cold they are encouraged to wear a face mask if they want…If the staff have any signs or symptoms then they would normally, they don’t really come to work, they would stay at home, but if they have like a cold and they’re over it when they come to us they will wear a face mask for a few more days. –* 042, W3 Staff, Intervention, Site Q

Our triangulation of this subtheme found *Agreement* since both the qualitative and quantitative datasets indicated that practices of and confidence in infection prevention and control strategies were not impacted by the intervention.

### Trial data collection practices

We found that there was variation in how study champions collected and inputted the data for the trial, but that monitoring residents’ symptoms and health was perceived to align with everyday care home duties.

Data on infection episodes for the primary outcome was collected by staff in the care homes. Between ten and sixteen residents at each home were randomly selected to give consent for access to their medical records and complete study questionnaires, in intervention homes these residents also gave consent to have a HEPA filter in their bedroom. Anonymised data on infection symptoms was collected on up to thirty residents in each home (including the up to sixteen who were answering questionnaires) by care home staff. All data was entered into the online AFRI-c database. Qualitative analysis identified three different ways in which staff collected this data. The first involved a staff member checking each resident and immediately inputting to the database. The second involved a staff member checking on residents throughout the day and inputting at the end of the day. The third involved a staff member using daily handovers or online care plans rather than individually checking with each resident. An example quote describing each of these approaches is below:

*We go in each patient’s room and…just look if they have like a runny nose or if they are touching their ear more or if they have vomiting or any temperature or anything like that, and then I record those.* – 042, W3 Staff, Intervention, Site Q

*I go round to all the residents every day, I ask them how they’re feeling, if they’ve got any symptoms of colds, any kind of things, chest infections etc. I ask them generally how they’re feeling. Then at the end of the day if nothing’s cropped up I’ll then sort of put in that everybody’s been alright.* – 016, W2 Staff, Usual care, Site E

*I’m quite happy watching the telly and uploading the info, because it’s nice and quiet and I can concentrate, and it doesn’t take all that long once you get into it. But yeah, I’ll sit there with a cup of tea and do it and then I can get on. I’ve usually got a list with me from the day, of everybody’s issues and I can go on the care plans live from home anyway and see what’s occurring*. – 020, W2 Staff, Usual Care, Site H

In the third approach, staff obtained information about multiple residents from verbal handovers or from online care systems which were updated by carers throughout the day. This approach relied on residents reporting symptoms, and/or other staff in the care home picking up on residents’ symptoms and sharing this at handovers or reporting it on their online system. This was by far the most commonly reported approach to data collection, perhaps unsurprisingly since it was the most time-efficient. However, mild symptoms or episodes could have been missed more frequently using this approach, since staff were not systematically asking each resident each question every day.

Checking on, or being aware of, the health of residents was seen as aligned with everyday care home staff duties. However, some reported finding the data collection easier to integrate it into their everyday role than others.

*I walk around and talk to residents anyway. That’s part of my role.* – 049, W3 Staff, Intervention, Site T

*I’d rather just sit down and just do it all in one hit, to be honest, when I’ve got like a spare five minutes than take it round ‘cause obviously you don’t know what each resident’s gonna be like and you don’t know the situation or their behaviour. –* 011, W1 Staff, Intervention, Site D

We might have expected that more senior staff (e.g., care home managers) had less time to directly check on residents, however some managers said they normally spent time with each resident daily and had time to input the data, whereas others did not have time at all at work, instead entering the data from home. Equally, some more junior carers integrated data collection directly from each resident into their rounds, whereas others felt they did not have time. This highlights the heterogeneity and complexity of care home settings.

As described earlier, staff were observing and reflecting on which residents were experiencing infections and forming an opinion on whether the HEPA filters were working or not. We explored how this might have influenced their data collection practices, but staff told us they would still report symptoms in a resident with a HEPA filter. As one staff member pointed out, reporting symptoms is an important part of their job within the home for the health and care of the residents, not only for the AFRI-c study.

*I would report [symptoms] no matter what even if they didn’t have an air filter ’cause they go down very quick if people are noticing and they’re not reporting it – something could have been done and something could have happened before things got worse. –* 001, W1 Staff, Intervention, Site A

Our triangulation of this meta-theme found *Silence* since trial data collection practices were not explored using quantitative methods.

## Discussion

### Intervention acceptability, fidelity, and perceived effectiveness

HEPA filters were generally acceptable to care home residents and staff, and self-reported compliance was high. Qualitative interviews highlighted challenges around keeping HEPA filters switched on, and how participants drew on their appraisal of infection rates but also odour and air quality when assessing if the HEPA filters were working.

The HEPA filters did not change satisfaction with care home environment or sleep quality. However, in care homes with small rooms, it may be difficult to position a HEPA filter far enough away from chairs or beds, to prevent residents feeling bothered by a cold draught. Portable standing HEPA filters were not considered suitable for every resident, and for those with cognitive impairment, could represent a safety hazard. Further findings about lessons learned regarding the use of such devices in this setting will be published elsewhere [19]. The HEPA filters did not interfere with everyday work and living in the care home, and people often forgot about them. In other words, minimal *cognitive participation* (engagement) was required to implement the intervention once installed. Staff sometimes reported needing to implement reminders to other staff not to turn off the HEPA filters. They also sometimes had to remind residents or regularly explain the purpose of the units. This was a form of *collective action* (steps taken to use HEPA filters), but it had minimal burden on the staff.

Little *reflexive monitoring* (appraisal of the HEPA filters) was needed to embed them in the care homes. Beyond the creation of signs and reminders to keep them switched on, the participants did not need to make adjustments to the HEPA filters. Staff appraised their effectiveness, drawing on their perceptions of number of infections in the care homes that winter. Some people felt that the HEPA filters had reduced infections, whereas others felt they had seen a similar number of infections as in previous winters. Interviewees also drew on perceptions of odour and air quality, to appraise effectiveness. Increased noise from the HEPA filters and seeing the material inside the filter when cleaning it, were also indicators to staff that they were working, and some staff felt ‘safer’ in rooms with HEPA filters. We did not collect quantitative data on staff sense of ‘safety’ since this was an unanticipated finding, but it is notable that this was not matched with statistically significant improvements in satisfaction with care home environment or sleep quality. Staff appreciated this sense of improved safety, but acknowledged that this may be a psychological construct and not reflect physical outcomes. Staff interviewed in Winter 1 (2021−2022) appeared to discuss feeling safer more than in subsequent winters. Rates of COVID-19 were at the highest during Winter 1 of the study (1 in 20 people in England had COVID-19) [20], and care home staff were still required to wear personal protective equipment and use lateral flow tests regularly. It is possible that as restrictions began to ease and concern about COVID-19 reduced, people felt less preoccupied with the possibility of airborne respiratory diseases. Belief that the HEPA filters would reduce infection transmission appeared to fall in the intervention group over time, but only by a small amount.

Most staff and residents agreed that infections could be transmitted through airborne particles, and that HEPA filters might reduce respiratory infections, indicating there was high *coherence* (understanding the purpose of HEPA filters). However, people had differing views on the need for HEPA filters in care homes. For staff, preventing infections was a top priority. For residents and some consultees, quality of life and care from staff was more important than preventing infections and prolonging life.

### Interpreting the AFRI-c clinical effectiveness findings

The AFRI-c study found no evidence that HEPA filters reduced winter respiratory infection episodes in residents (with and without bedroom HEPA filters), or staff absence. A lower-than-expected infection rate was seen across both arms [11]. Unlike the HEPA filters themselves, the data collection for AFRI-c did require cognitive engagement from staff. The most commonly reported approach to data collection was to consult online care plans and databases to retrieve the data, rather than directly assessing residents. This approach was more time-efficient for busy staff, but it may have meant that episodes of infection were missed, since milder symptoms may not have been reported to staff, or if symptoms were not added to the care home online system or mentioned during verbal handovers. It is possible that such episodes were under-reported in the control arm, compared with the intervention arm, because the HEPA filters may have acted as a prompt in the intervention homes to collect the daily data directly from residents (staff were also being asked to report daily whether the HEPA filters were in position and switched on). If this had occurred, it would have led to the intervention appearing less effective. That said, care home staff are aware that reporting even mild symptoms is important because they can lead to serious illness in residents with low function reserve. Intervention adherence (i.e., HEPA filters switched on at least 20% of the time) was extremely high and was not significantly correlated with infection outcomes, but this was self-reported and staff separately described incidents of residents and other staff mistakenly switching off HEPA filters. The quantitative data indicates some use of ‘non-trial’ HEPA filters, particularly amongst residents in intervention homes who were not invited to have a bedroom HEPA filter (known as ‘communal room HEPA filter residents’). This provides further evidence of intervention acceptability and is unlikely to have undermined our null finding. We did not find evidence that the HEPA filters impacted on the other infection prevention and control strategies carried out routinely by the care homes.

### Comparison with existing literature

Other research has found that the cold draught from air purifiers is a challenge for users, with one study finding a statistically significant decrease in use of air purifiers during cooler periods [21], and a systematic review of 148 studies of the effectiveness of portable air cleaners finding that occupants used the air cleaners for shorter periods and on low airflow rate settings due in part to concerns about impact on temperature [22]. Another study reported that noise from portable air filters was not considered a problem by participants [23].

In a systematic review and synthesis of care home trial process evaluations, the most important contextual factor identified was the compatibility of the intervention with existing work practices [8]. In AFRI-c, the intervention did not substantially affect everyday life and work within the home and people often forgot they were there, which is a strength. The same review identified a key issue of procedural drift, as studies tended to lose momentum over time, and deviations to the intended protocol crept in. While we did not identify significant deviations to the intervention protocol, practices of data collection did seem to veer from the original plan over time, in that residents were not being asked about specific symptoms. For some staff the data collection was not easily integrated into their practice, and this led to approaches which could have resulted in missing milder symptoms and episodes of respiratory infections. A strength of the AFRI-c trial was that the implementation period was relatively short at each site (one winter period, usually up to 9 months), which may have mitigated procedural drift to some extent. Others have noted the challenges of introducing innovations into care homes where the benefits are not immediate or clear, and where outcomes occur in a complex system [24]. This unclear link between outcomes and intervention may mean that a trial or intervention is viewed less positively and reduce cognitive engagement with the study. In our study, minimal cognitive engagement with the intervention was required, but some staff described challenges in fitting data collection into their everyday work. Future studies requiring primary outcome data collection by care home staff could implement participatory activities that emphasise the importance of adhering to research protocols, targeted at study champions. They could also try to ensure before starting that staff truly have protected time to conduct study processes [25], although we recognise that this is challenging in the care home sector.

### Strengths and limitations

We were able to include the voices of residents in Winters 2 and 3, which provided important data for our analysis. COVID-19 outbreaks and the need to conduct interviews in person to enhance engagement, meant that we were unable to interview residents in Winter 1. This may have yielded different views on infections and the intervention, given that care homes were still in pandemic restrictions at that time. Staff questionnaires were optional, so selection bias may be present, and the low response rate limited our ability to conduct regression analyses as planned. To reduce staff burden, we did not collect baseline characteristics of staff who were involved in AFRI-c or who completed the questionnaires, which makes it challenging to understand if our sample of questionnaire respondents represents the staff involved in AFRI-c. While we purposively sampled sites to collect resident interview data from, we were unable to purposively sample residents within these sites, because we were limited to those who agreed to be approached and who had capacity to consent. We recommend that future research utilises objective measures of adherence, such as air quality monitors. An ethnographic approach to the process evaluation may have also yielded further useful insights about, e.g., adherence and interactions with the intervention.

## Conclusions

Our mixed methods process evaluation of the AFRI-c trial found the use of HEPA filters was acceptable, with high levels of compliance and low levels of contamination. The filters became normalised in the care homes, although they were not considered suitable for every resident and some residents were affected by the cold draught. Some effort to ensure they were kept on was required. It is possible that the approach to data collection caused under-reporting of mild infections. We should not assume that infection prevention is always a priority, particularly for residents.

## Supporting information

S1 Table Characteristics of care homes included in the qualitative study. ^*^Index of Multiple Deprivation score, 1 = most deprived, 10 = least deprived; ^±^Only care homes with a CQC rating of Good or Outstanding were eligible for AFRI-c.
(DOCX)

S2 Table Demographics of interviewed participants. (DOCX)

S3 Table Baseline characteristics of active residents in AFRI-c. ^1^Median and Interquartile range; ^2^Collected at resident screening (registry).
(DOCX)

S4 Table Resident/consultee satisfaction with care home environment. (DOCX)

S5 Table Staff satisfaction with care home environment. (DOCX)

S6 Table Resident/consultee beliefs. (DOCX)

S7 Table Staff beliefs and confidence. (DOCX)

S8 Table Number of patients and days with a new non-trial HEPA filter installed in a bedroom. (DOCX)

S9 Table Binary compliance in the bedroom HEPA filter population. ^±^Compliance was defined as the HEPA filters in position and switched on at least 20% of the time (daily data recorded by staff).
(DOCX)

S10 Table Continuous compliance in the bedroom HEPA filter population. ^±^Compliance was defined as the HEPA filters in position and switched on at least 20% of the time (daily data recorded by staff).
(DOCX)

S11 Table Binary compliance in the communal room HEPA filter population. ^±^Compliance was defined as the HEPA filters in position and switched on at least 20% of the time (daily data recorded by staff).
(DOCX)

S12 Table Continuous compliance in the communal room HEPA filter population. ^±^Compliance was defined as the HEPA filters in position and switched on at least 20% of the time (daily data recorded by staff).
(DOCX)
