# Supplementary material for: Implementation and acceptability of high efficiency particulate air filters to reduce respiratory infections in care homes: Process evaluation of the AFRI-c cluster randomised controlled trial
Source: PLoS One. 2026 Jul 27;21(7):e0347989. doi: 10.1371/journal.pone.0347989 (PMC13405086; doi:10.1371/journal.pone.0347989)
Supplement: S1 Table — *Index of Multiple Deprivation score, 1 = most deprived, 10 = least deprived; ±Only care homes with a CQC rating of Good or Outstanding were eligible for AFRI-c. (DOCX) [file pone.0347989.s001.docx]

**S1 Table – Characteristics of care homes included in the qualitative study**

| **Care home characteristic** | **Number (%)** |
| --- | --- |
| IMD decile*  1-5  6-10  *Mean* | 10 (45%)  12 (55%)  *6* |
| English Region  South West  South East  West Midlands  North East  North West  East of England | 9 (41%)  2 (9%)  4 (18%)  3 (14%)  1 (4%)  3 (14%) |
| Size (maximum capacity)  20-40  41-60  61+  *Mean size*  *Range* | 14 (64%)  5 (22%)  3 (14%)  *41*  *25-73* |
| Care Quality Commission (CQC)± rating (at baseline)  Good  Outstanding | 21 (95%)  1 (5%) |
| Nursing care offered  Yes | 8 (36%) |
| Dementia care offered  Yes | 6 (27%) |
| Part of a chain  Yes | 10 (45%) |
| Trial arm  Intervention  Control | 17 (77%)  5 (23%) |
| Winter  1  2  3 | 4 (18%)  10 (46%)  8 (36%) |
| **TOTAL** | **22** |

*Index of Multiple Deprivation score, 1=most deprived, 10=least deprived;

±Only care homes with a CQC rating of Good or Outstanding were eligible for AFRI-c
