# Supplementary material for: Implementation and acceptability of high efficiency particulate air filters to reduce respiratory infections in care homes: Process evaluation of the AFRI-c cluster randomised controlled trial
Source: PLoS One. 2026 Jul 27;21(7):e0347989. doi: 10.1371/journal.pone.0347989 (PMC13405086; doi:10.1371/journal.pone.0347989)
Supplement: S2 Table — (DOCX) [file pone.0347989.s002.docx]

**S2 Table - Demographics of interviewed participants**

| **Participant** | **Characteristic** | **Number (%)** |
| --- | --- | --- |
| **Staff** | Role  Manager  Deputy Manager  Carer/Senior Carer  Activities Manager | 13 (52%)  6 (24%)  5 (20%)  1 (6%) |
|  | Length of experience  1-5 years  6-10 years  11-15 years  16-20 years  >20 years | 6 (24%)  2 (8%)  3 (12%)  2 (8%)  12 (48%) |
|  | Nurse/carer background  Yes  No | 20 (80%)  5 (20%) |
|  | Care home providing nursing care  Yes  No | 10 (40%)  15 (60%) |
|  | Winter  1  2  3 | 7 (28%)  11 (44%)  7 (28%) |
|  | Study arm  Intervention  Usual care | 18 (72%)  7 (28%) |
|  | **Total** | **25 (44% of all interviewees)** |
| **Residents** | Age  50-59  60-69  70-79  80-89  90-99 | 1 (5%)  1 (5%)  4 (20%)  4 (20%)  10 (50%) |
|  | Gender  Female  Male | 13 (65%)  7 (35%) |
|  | Ethnicity  White | 20 (100%) |
|  | Type of care  Residential  Nursing care | 19 (95%)  1 (5%) |
|  | Winter  1  2  3 | 0 (0)  11 (55%)  9 (45%) |
|  | **Total** | **20 (35% of all interviewees)** |
| **Consultees** | Relationship to resident  Daughter  Daughter-in-law  Son  Stepdaughter  Wife | 1 (8%)  1 (8%)  5 (42%)  2 (17%)  3 (25%) |
|  | Gender of resident  Female  Male | 7 (58%)  5 (42%) |
|  | Ethnicity of resident  White | 12 (100%) |
|  | Resident in receipt of nursing care  Yes  No | 7 (58%)  5 (42%) |
|  | Winter  1  2  3 | 4 (33%)  2 (17%)  6 (50%) |
|  | **Total** | **12 (21% of all interviewees)** |
| **ALL** | **TOTAL** | **57** |
