# Supplementary material for: Implementation and acceptability of high efficiency particulate air filters to reduce respiratory infections in care homes: Process evaluation of the AFRI-c cluster randomised controlled trial
Source: PLoS One. 2026 Jul 27;21(7):e0347989. doi: 10.1371/journal.pone.0347989 (PMC13405086; doi:10.1371/journal.pone.0347989)
Supplement: S3 Table — 1Median and Interquartile range; 2Collected at resident screening (registry). (DOCX) [file pone.0347989.s003.docx]

**S3 Table - Baseline characteristics of active residents in AFRI-c**

|  | **Intervention** | **Control** | **Overall** |
| --- | --- | --- | --- |
|  | **N = 569** | **N = 589** | **N = 1158** |
| Sex (male) | 171 / 569 (30%) | 166 / 589 (28%) | 337 / 1158 (29%) |
| Age (years)^1^ | 87 (81, 92) | 88 (82, 92) | 88 (82, 92) |
| Ethnicity: |  |  |  |
| White or Caucasian | 564 / 568 (99%) | 582 / 589 (99%) | 1146 / 1157 (99%) |
| Black/African/Caribbean/ Black British | 3 / 568 (1%) | 6 / 589 (1.0%) | 9 / 1157 (1%) |
| Asian/Asian British | 0 / 568 (0%) | 1 / 589 (0.2%) | 1 / 1157 (0.1%) |
| Other ethnic groups | 1 / 568 (0%) | 0 / 589 (0%) | 1 / 1157 (0.1%) |
| Receives nursing care | 199 / 568 (35%) | 253 / 589 (43%) | 452 / 1157 (39%) |
| Has dementia^2^ | 333 / 569 (59%) | 332 / 589 (56%) | 665 / 1158 (57%) |
| Frailty score^1, 2^ | 6 (4, 7) | 6 (5, 7) | 6 (4, 7) |
| Has received Influenza vaccine^2^ | 538 / 569 (95%) | 554 / 589 (94%) | 1092 / 1158 (94%) |
| Has received COVID-19 vaccine^2^ | 553 / 569 (97%) | 562 / 588 (96%) | 1115 / 1157 (96%) |

^1^Median and Interquartile range; ^2^Collected at resident screening (registry)
