# Supplementary material for: Implementation and acceptability of high efficiency particulate air filters to reduce respiratory infections in care homes: Process evaluation of the AFRI-c cluster randomised controlled trial
Source: PLoS One. 2026 Jul 27;21(7):e0347989. doi: 10.1371/journal.pone.0347989 (PMC13405086; doi:10.1371/journal.pone.0347989)
Supplement: S4 Table — (DOCX) [file pone.0347989.s004.docx]

**S4 Table – Resident/consultee satisfaction with care home environment**

|  | **BASELINE** | |  | **FOLLOW-UP** | | |
| --- | --- | --- | --- | --- | --- | --- |
|  | **Intervention** | **Control** | **Total** | **Intervention** | **Control** | **Total** |
| **Perceptions of care home environment temperature** | | | | | | |
| Very satisfied | 140 (39.3%) | 166 (42.2%) | **306 (40.9%)** | 147 (41.3%) | 182 (46.3%) | **329 (43.9%)** |
| Satisfied | 190 (53.4%) | 197 (50.1%) | **387 (51.7%)** | 176 (49.4%) | 167 (42.5%) | **343 (45.8%)** |
| Not sure | 16 (4.5%) | 8 (2%) | **24 (3.2%)** | 17 (4.8%) | 19 (4.8%) | **36 (4.8%)** |
| Dissatisfied | 9 (2.5%) | 19 (4.8%) | **28 (3.7%)** | 16 (4.5%) | 19 (4.8%) | **35 (4.7%)** |
| Very dissatisfied | 1 (0.3%) | 3 (0.8%) | **4 (0.5%)** | 0 (0%) | 6 (1.5%) | **6 (0.8%)** |
| **Overall** | **356 (100%)** | 393 (100%) | **749 (100%)** | **356 (100%)** | **393 (100%)** | **749 (100%)** |
| **Perceptions of care home environment odour** | | | | | | |
| Very satisfied | 131 (37%) | 189 (48.1%) | **320 (42.8%)** | 188 (53.1%) | 182 (46.3%) | **391 (52.3%)** |
| Satisfied | 188 (53.1%) | 176 (44.8%) | **364 (48.7%)** | 135 (38.1%) | 167 (42.5%) | **282 (37.8%)** |
| Not sure | 25 (7.1%) | 24 (6.1%) | **49 (6.6%)** | 25 (7.1%) | 19 (4.8%) | **58 (7.8%)** |
| Dissatisfied | 9 (2.5%) | 3 (0.8%) | **12 (1.6%)** | 6 (1.7%) | 19 (4.8%) | **14 (1.9%)** |
| Very dissatisfied | 1 (0.3%) | 1 (0.3%) | **2 (0.3%)** | 0 (0%) | 6 (1.5%) | **2 (0.3%)** |
| **Overall** | **354 (100%)** | **393 (100%)** | **747 (100%)** | **354 (100%)** | **393 (100%)** | **747 (100%)** |
| **Perceptions of care home environment air quality** | | | | | | |
| Very satisfied | 96 (27%) | 110 (28%) | **206 (27.5%)** | 141 (39.6%) | 140 (35.6%) | **281 (37.5%)** |
| Satisfied | 200 (56.2%) | 217 (55.2%) | **417 (55.7%)** | 168 (47.2%) | 189 (48.1%) | **357 (47.7%)** |
| Not sure | 49 (13.8%) | 62 (15.8%) | **111 (14.8%)** | 43 (12.1%) | 56 (14.2%) | **99 (13.2%)** |
| Dissatisfied | 11 (3.1%) | 4 (1%) | **15 (2%)** | 4 (1.1%) | 6 (1.5%) | **10 (1.3%)** |
| Very dissatisfied | 0 (0%) | 0 (0%) | **0 (0%)** | 0 (0%) | 2 (0.5%) | **2 (0.3%)** |
| **Overall** | **356 (100%)** | **393 (100%)** | **749 (100%)** | **356 (100%)** | **393 (100%)** | **749 (100%)** |
| **Sleep quality satisfaction** | | | | | | |
| Very satisfied | 86 (24.5%) | 113 (28.8%) | **199 (26.9%)** | 114 (32.5%) | 132 (33.6%) | **246 (33.2%)** |
| Satisfied | 147 (41.9%) | 166 (42.2%) | **313 (42.2%)** | 140 (39.9%) | 163 (41.5%) | **303 (40.9%)** |
| Not sure | 76 (21.7%) | 65 (16.5%) | **141 (19%)** | 70 (19.9%) | 53 (13.5%) | **123 (16.6%)** |
| Dissatisfied | 25 (7.1%) | 35 (8.9%) | **60 (8.1%)** | 20 (5.7%) | 32 (8.1%) | **52 (7%)** |
| Very dissatisfied | 17 (4.8%) | 11 (2.8%) | **28 (3.8%)** | 7 (2%) | 10 (2.5%) | **17 (2.3%)** |
| **Overall** | **351 (100%)** | **393 (100%)** | **741 (100%)** | **351 (100%)** | **393 (100%)** | **741 (100%)** |
