# Supplementary material for: Implementation and acceptability of high efficiency particulate air filters to reduce respiratory infections in care homes: Process evaluation of the AFRI-c cluster randomised controlled trial
Source: PLoS One. 2026 Jul 27;21(7):e0347989. doi: 10.1371/journal.pone.0347989 (PMC13405086; doi:10.1371/journal.pone.0347989)
Supplement: S5 Table — (DOCX) [file pone.0347989.s005.docx]

**S5 Table - Staff satisfaction with care home environment**

|  | **BASELINE** | |  |  | **FOLLOW-UP** | |  |
| --- | --- | --- | --- | --- | --- | --- | --- |
|  | **Intervention** | **Control** | **Total** |  | **Intervention** | **Control** | **Total** |
|  | **Perception of care home environment temperature** | | | | | | |
| Very satisfied | 29 (27.1%) | 26 (22.8%) | **55 (24.9%)** |  | 72 (39.8%) | 47 (28.1%) | **119 (34.2%)** |
| Satisfied | 60 (56.1%) | 64 (56.1%) | **124 (56.1%)** |  | 82 (45.3%) | 95 (56.9%) | **177 (50.9%)** |
| Not sure | 9 (8.4%) | 17 (14.9%) | **26 (11.8%)** |  | 13 (7.2%) | 12 (7.2%) | **25 (7.2%)** |
| Dissatisfied | 8 (7.5%) | 5 (4.4%) | **13 (5.9%)** |  | 13 (7.2%) | 9 (5.4%) | **22 (6.3%)** |
| Very dissatisfied | 1 (0.9%) | 2 (1.8%) | **3 (1.4%)** |  | 1 (0.6%) | 4 (2.4%) | **5 (1.4%)** |
| **Overall** | **107 (100%)** | **114 (100%)** | **221 (100%)** |  | **181 (100%)** | **167 (100%)** | **348 (100%)** |
|  | **Perception of care home environment odour** | | | | | | |
| Very satisfied | 40 (37.4%) | 36 (31.9%) | **76 (34.6%)** |  | 68 (37.8%) | 72 (43.6%) | **140 (40.6%)** |
| Satisfied | 54 (50.5%) | 53 (46.9%) | **107 (48.6%)** |  | 92 (51.1%) | 75 (45.6%) | **167 (48.4%)** |
| Not sure | 9 (8.4%) | 12 (10.6%) | **21 (9.6%)** |  | 12 (6.7%) | 16 (9.7%) | **28 (8.1%)** |
| Dissatisfied | 4 (3.7%) | 12 (10.6%) | **16 (7.3%)** |  | 6 (3.3%) | 1 (0.6%) | **7 (2.0%)** |
| Very dissatisfied | 0 | 0 | **0** |  | 2 (1.1%) | 1 (0.6%) | **3 (0.9%)** |
| **Overall** | **107 (100%)** | **113 (100%)** | **220 (100%)** |  | **180 (100%)** | **165 (100%)** | **345 (100%)** |
|  | **Perception of care home environment air quality** | | | | | | |
| Very satisfied | 30 (28.3%) | 25 (22.3%) | **55 (25.2%)** |  | 61 (34.3%) | 47 (28.5%) | **108 (31.5%)** |
| Satisfied | 54 (50.9%) | 51 (45.5%) | **105 (48.2%)** |  | 94 (52.8%) | 93 (56.4%) | **187 (54.5%)** |
| Not sure | 15 (14.2%) | 24 (21.4%) | **39 (17.9%)** |  | 22 (12.4%) | 18 (10.9%) | **40 (11.74%)** |
| Dissatisfied | 6 (5.7%) | 9 (8.0%) | **15 (6.9%)** |  | 1 (0.6%) | 4 (2.4%) | **5 (1.5%)** |
| Very dissatisfied | 1 (0.9%) | 3 (2.7%) | **4 (1.8%)** |  | 0 | 3 (1.8%) | **3 (0.9%)** |
| **Overall** | **106 (100%)** | **112 (100%)** | **218 (100%)** |  | **178 (100%)** | **165 (100%)** | **343 (100%)** |
