# Supplementary material for: Implementation and acceptability of high efficiency particulate air filters to reduce respiratory infections in care homes: Process evaluation of the AFRI-c cluster randomised controlled trial
Source: PLoS One. 2026 Jul 27;21(7):e0347989. doi: 10.1371/journal.pone.0347989 (PMC13405086; doi:10.1371/journal.pone.0347989)
Supplement: S6 Table — (DOCX) [file pone.0347989.s006.docx]

**S6 Table – Resident/consultee beliefs**

|  | **BASELINE** | |  |  | **FOLLOW-UP** | | |
| --- | --- | --- | --- | --- | --- | --- | --- |
|  | **Intervention** | **Control** | **Total** |  | **Intervention** | **Control** | **Total** |
|  | **I believe infections can be spread through the air, for example as droplets from a sneeze or cough** | | | | | | |
| Strongly agree | 263 (74.1%) | 291 (74%) | **554 (74.1%)** |  | 251 (70.7%) | 274 (69.7%) | **525 (70.2%)** |
| Slightly agree | 36 (10.1%) | 55 (14%) | **91 (12.2%)** |  | 46 (13%) | 67 (17%) | **113 (15.1%)** |
| Not sure | 28 (7.9%) | 16 (4.1%) | **44 (5.9%)** |  | 20 (5.6%) | 19 (4.8%) | **39 (5.2%)** |
| Slightly disagree | 13 (3.7%) | 6 (1.5%) | **19 (2.5%)** |  | 8 (2.3%) | 5 (1.3%) | **13 (1.7%)** |
| Strongly disagree | 15 (4.2%) | 25 (6.4%) | **40 (5.3%)** |  | 30 (8.5%) | 28 (7.1%) | **58 (7.8%)** |
| **Overall** | **355 (100%)** | **393 (100%)** | **748 (100%)** |  | **355 (100%)** | **393 (100%)** | **748 (100%)** |
|  | **I believe air filters reduce infections being spread through the air** | | | | | | |
| Strongly agree | 90 (25.3%) | 80 (20.4%) | **170 (22.7%)** |  | 105 (29.5%) | 94 (23.9%) | **199 (26.6%)** |
| Slightly agree | 95 (26.7%) | 117 (29.8%) | **212 (28.3%)** |  | 101 (28.4%) | 126 (32.1%) | **227 (30.3%)** |
| Not sure | 156 (43.8%) | 178 (45.3%) | **334 (44.6%)** |  | 116 (32.6%) | 146 (37.2%) | **262 (35%)** |
| Slightly disagree | 13 (3.7%) | 4 (1%) | **17 (2.3%)** |  | 17 (4.8%) | 10 (2.5%) | **27 (3.6%)** |
| Strongly disagree | 2 (0.6%) | 14 (3.6%) | **16 (2.1%)** |  | 17 (4.8%) | 17 (4.3%) | **34 (4.5%)** |
| **Overall** | **356 (100%)** | **393 (100%)** | **749 (100%)** |  | **356 (100%)** | **393 (100%)** | **749 (100%)** |
