# Supplementary material for: Implementation and acceptability of high efficiency particulate air filters to reduce respiratory infections in care homes: Process evaluation of the AFRI-c cluster randomised controlled trial
Source: PLoS One. 2026 Jul 27;21(7):e0347989. doi: 10.1371/journal.pone.0347989 (PMC13405086; doi:10.1371/journal.pone.0347989)
Supplement: S7 Table — (DOCX) [file pone.0347989.s007.docx]

**S7 Table - Staff beliefs and confidence**

|  |  | **BASELINE** |  |  | **FOLLOW-UP** | |  |
| --- | --- | --- | --- | --- | --- | --- | --- |
|  | **Intervention** | **Control** | **Total** |  | **Intervention** | **Control** | **Total** |
|  | **I know how to prevent infection transmission** | | | | | | |
| Strongly agree | 92 (86.0%) | 82 (70.7%) | **174 (78.0%)** |  | 148 (80.9%) | 151 (89.9%) | **299 (85.2%)** |
| Slightly agree | 11 (10.3%) | 24 (20.7%) | **35 (15.7%)** |  | 20 (10.9%) | 7 4.2%) | **27 (7.7%)** |
| Not sure | 2 (1.9%) | 4 (3.5%) | **6 (2.7%)** |  | 2 (1.1%) | 2 (1.2%) | **4 (1.1%)** |
| Slightly disagree | 0 | 1 (0.9%) | **1 (0.5%)** |  | 2 (1.1%) | 0 | **2 (0.6%)** |
| Strongly disagree | 2 (1.9%) | 5 (4.3%) | **7 (3.1%)** |  | 11 (6.0%) | 8 (4.8%) | **19 (5.4%)** |
| **Overall** | **107 (100%)** | **116 (100%)** | **223 (100%)** |  | **183 (100%)** | **168 (100%)** | **351 (100%)** |
|  | **Lack of time/facilities sometimes prevents me from following infection control procedures** | | | | | | |
| Strongly agree | 10 (9.4%) | 10 (8.6%) | **20 (9.0%)** |  | 12 (6.6%) | 18 (10.7%) | **30 (8.6%)** |
| Slightly agree | 18 (16.8%) | 28 (24.1%) | **46 (20.6%)** |  | 37 (20.2%) | 29 (17.3%) | **66 (18.8%)** |
| Not sure | 4 (3.7%) | 11 (9.5%) | **15 (6.7%)** |  | 6 (3.3%) | 4 (2.4%) | **10 (2.9%)** |
| Slightly disagree | 13 (12.2%) | 21 (18.1%) | **34 (15.3%)** |  | 30 (16.4%) | 27 (16.1%) | **57 (16.2%)** |
| Strongly disagree | 62 (57.9%) | 46 (39.7%) | **108 (48.4%)** |  | 98 (53.6%) | 90 (53.6%) | **188 (53.6%)** |
| **Overall** | **107 (100%)** | **116 (100%)** | **223 (100%)** |  | **183 (100%)** | **168 (100%)** | **351 (100%)** |
|  | **I am confident my use of infection procedures is effective** | | | | | | |
| Strongly agree | 92 (86.0%) | 75 (64.7%) | **167 (74.9%)** |  | 156 (85.3%) | 148 (88.6%) | **304 (86.9%)** |
| Slightly agree | 9 (8.4%) | 28 (24.1%) | **37 (16.6%)** |  | 15 (8.2%) | 9 (5.4%) | **24 (6.9%)** |
| Not sure | 2 (1.9%) | 5 (4.3%) | **7 (3.1%)** |  | 2 (1.1%) | 4 (2.4%) | **6 (1.7%)** |
| Slightly disagree | 0 | 2 (1.7%) | **2 (0.9%)** |  | 1 (0.6%) | 1 (0.6%) | **2 (0.6%)** |
| Strongly disagree | 4 (3.7%) | 6 (5.2%) | **10 (4.5%)** |  | 9 (4.9%) | 5 (3.0%) | **14 (4.0%)** |
| **Overall** | **107 (100%)** | **116 (100%)** | **223 (100%)** |  | **183 (100%)** | **167 (100%)** | **350 (100%)** |
|  | **I believe infections can be spread through the air, for example as droplets from a sneeze or cough** | | | | | | |
| Strongly agree | 96 (89.7%) | 95 (81.9%) | **191 (85.7%)** |  | 157 (86.3%) | 149 (88.7%) | **306 (87.4%)** |
| Slightly agree | 7 (6.5%) | 15 (12.9%) | **22 (9.9%)** |  | 15 (8.2%) | 11 (6.6%) | **26 (7.4%)** |
| Not sure | 1 (0.9%) | 2 (1.7%) | **3 (1.4%)** |  | 1 (0.6%) | 3 (1.8%) | **4 (1.1%)** |
| Slightly disagree | 0 | 3 (2.6%) | **3 (1.4%)** |  | 0 | 1 (0.6%) | **1 (0.3%)** |
| Strongly disagree | 3 (2.8%) | 1 (0.9%) | **4 (1.8%)** |  | 9 (5.0%) | 4 (2.4%) | **13 (3.7%)** |
| **Overall** | **107 (100%)** | **116 (100%)** | **223 (100%)** |  | **182 (100%)** | **168 (100%)** | **350 (100%)** |
|  | **I believe air filters reduce infections being spread through the air** | | | | | | |
| Strongly agree | 34 (31.8%) | 24 (21.1%) | **58 (26.2%)** |  | 87 (48.3%) | 54 (34.6%) | **141 (42.0%)** |
| Slightly agree | 35 (32.7%) | 44 (38.6%) | **79 (35.8%)** |  | 45 (25.0%) | 35 (22.4%) | **80 (23.8%)** |
| Not sure | 36 (33.6%) | 38 (33.3%) | **74 (33.5%)** |  | 30 (16.7%) | 58 (37.2%) | **88 (26.2%)** |
| Slightly disagree | 1 (0.9%) | 4 (3.5%) | **5 (2.3%)** |  | 11 (6.1%) | 6 (3.9%) | **17 (5.1%)** |
| Strongly disagree | 1 (0.9%) | 4 (3.5%) | **5 (2.3%)** |  | 7 (3.9%) | 3 (1.9%) | **10 (3.0%)** |
| **Overall** | **107 (100%)** | **114 (100%)** | **221 (100%)** |  | **180 (100%)** | **156 (100%)** | **336 (100%)** |
