# Supplementary material for: Implementation and acceptability of high efficiency particulate air filters to reduce respiratory infections in care homes: Process evaluation of the AFRI-c cluster randomised controlled trial
Source: PLoS One. 2026 Jul 27;21(7):e0347989. doi: 10.1371/journal.pone.0347989 (PMC13405086; doi:10.1371/journal.pone.0347989)
Supplement: S8 Table — (DOCX) [file pone.0347989.s008.docx]

**S8 Table - Number of patients and days with a new non-trial HEPA filter installed in a bedroom**

| Allocation | Residents with non-trial filter in bedroom^1^ | Days non-trial filters on in private rooms^2^ | Days with missing air filter data^3^ |
| --- | --- | --- | --- |
| *BEDROOM HEPA FILTER RESIDENTS* | | | |
| Intervention^3^ | 15 / 569 (2.6%) | 45 / 96834 (0.05%) | 0 / 96834 (0%) |
| Control | 21 / 589 (3.6%) | 30 / 104485 (0.03%) | 2698 / 104485 (2.6%) |
| **Overall** | **36 / 1158 (3.1%)** | **75 / 201319 (0.04%)** | **2698 / 201319 (1.3%)** |
| *COMMUNAL ROOM HEPA FILTER RESIDENTS* | | | |
| Intervention | 176 / 1004 (17.5%) | 1466 / 127785 (1.2%) | 11396 / 127785 (8.9%) |
| Control | 37 / 932 (4.0%) | 58 / 130501 (0.04%) | 7467 / 130501 (5.7%) |
| **Overall** | **213 / 1936 (11.0%)** | **18863 / 258286 (0.59%)** | **18863 / 258286 (7.3%)** |

^1^Denominators are total number of participants in allocation and population

^2^Denominators are total number of valid days. Valid days are defined as resident follow-up days with daily data collection, excluding days when a resident’s data is not required or a resident is not in the care home, e.g., hospitalised, other medical appointment.

^3^Missing days are counted as zero as bedroom HEPA filter residents in the intervention arm were not assessed for non-trial air filters being present in their rooms
