# Supplementary material for: Implementation and acceptability of high efficiency particulate air filters to reduce respiratory infections in care homes: Process evaluation of the AFRI-c cluster randomised controlled trial
Source: PLoS One. 2026 Jul 27;21(7):e0347989. doi: 10.1371/journal.pone.0347989 (PMC13405086; doi:10.1371/journal.pone.0347989)
Supplement: S10 Table — ±Compliance was defined as the HEPA filters in position and switched on at least 20% of the time (daily data recorded by staff). (DOCX) [file pone.0347989.s012.docx]

**S12 Table - Continuous Compliance in the Communal Room HEPA Filter Population**

| **Winter** | **Median (IQR)** |
| --- | --- |
| 1 | 87.7% (79.8%, 87.7%) |
| 2 | 95.9% (87.9%, 98.8%) |
| 3 | 97.3% (93.2%, 98.2%) |
| **Overall** | **95.9% (87.7%, 98.2%)** |

± Compliance was defined as the HEPA filters in position and switched on at least 20% of the time (daily data recorded by staff)
