# Supplementary material for: Implementation and acceptability of high efficiency particulate air filters to reduce respiratory infections in care homes: Process evaluation of the AFRI-c cluster randomised controlled trial
Source: PLoS One. 2026 Jul 27;21(7):e0347989. doi: 10.1371/journal.pone.0347989 (PMC13405086; doi:10.1371/journal.pone.0347989)
Supplement: S11 Table — ±Compliance was defined as the HEPA filters in position and switched on at least 20% of the time (daily data recorded by staff). (DOCX) [file pone.0347989.s010.docx]

**S10 Table – Continuous Compliance in the Bedroom HEPA Filter Population**

| **Winter** | **Median (IQR) Compliance** |
| --- | --- |
| 1 | 80% (71.1%, 88.9%) |
| 2 | 97.6% (89.4%, 98.8%) |
| 3 | 97.1% (94.0%, 97.8%) |
| **Overall** | **96.8% (88.9%, 98.1%)** |

± Compliance was defined as the HEPA filters in position and switched on at least 20% of the time (daily data recorded by staff)
